# Milk consumption during pregnancy increases birth weight, a risk factor for the development of diseases of civilization

**DOI:** 10.1186/s12967-014-0377-9

**Published:** 2015-01-16

**Authors:** Bodo C Melnik, Swen Malte John, Gerd Schmitz

**Affiliations:** Department of Dermatology, Environmental Medicine and Health Theory, University of Osnabrück, Sedanstrasse 115, D-49090 Osnabrück, Germany; Institute of Clinical Chemistry and Laboratory Medicine, University Clinics of Regensburg, Regensburg, Germany

**Keywords:** Birth weight, Exosomal microRNA, Fetal weight, Gestational weight, Milk, mTORC1, Placental weight, Primary prevention

## Abstract

Antenatal dietary lifestyle intervention and nutrition during pregnancy and early postnatal life are important for appropriate lifelong metabolic programming. Epidemiological evidence underlines the crucial role of increased birth weight as a risk factor for the development of chronic diseases of civilization such as obesity, diabetes and cancer. Obstetricians and general practitioners usually recommend milk consumption during pregnancy as a nutrient enriched in valuable proteins and calcium for bone growth. However, milk is not just a simple nutrient, but has been recognized to function as an endocrine signaling system promoting anabolism and postnatal growth by activating the nutrient-sensitive kinase mTORC1. Moreover, pasteurized cow’s milk transfers biologically active exosomal microRNAs into the systemic circulation of the milk consumer apparently affecting more than 11 000 human genes including the mTORC1-signaling pathway. This review provides literature evidence and evidence derived from translational research that milk consumption during pregnancy increases gestational, placental, fetal and birth weight. Increased birth weight is a risk factor for the development of diseases of civilization thus involving key disciplines of medicine. With regard to the presented evidence we suggest that dietary recommendations promoting milk consumption during pregnancy have to be re-evaluated.

## Introduction

To meet the requirements for macronutrients and minerals during pregnancy gynecologists and general practitioners recommend increased servings of milk and dairy products [1]. Although milk is a rich source of essential amino acids and calcium, recent understanding of milk’s biological function has changed during the last years. Milk is not just a nutrient, but represents an endocrine signaling system of mammals activating the key regulator of cell growth and anabolism, the nutrient-sensitive kinase mTORC1 (*mechanistic target of rapamycin complex 1*) [2]. At the molecular level, cell growth, proliferation, and anabolism are regulated by mTORC1 [3-12]. In the perspective of human evolution, persistent cow’s milk consumption is a novel human behavior, which may result in long-term adverse health effects [13]. Increased mTORC1 signaling during milk consumption has recently been confirmed in a rodent model and has been associated with the development of obesity [14-17]. Pregravid maternal overweight and obesity are well-known risk factors that promote fetal overnutrition and fetal macrosomia [18-26]. Increased birth weight is a risk factor for the development of diseases of civilization, especially obesity [22,23,25]. To understand the impact of milk consumption on fetal growth, it is of critical importance to appreciate milk’s biological function as an activator of mTORC1 and transmitter of gene-regulatory exosomal microRNAs [2].

### Pivotal mTORC1-activating signals

mTORC1 orchestrates cell growth and proliferation [3]. mTORC1 is the central hub of metabolism that activates nucleotide, protein and lipid synthesis under conditions of nutrient and growth factor availability [3-12]. mTORC1 plays a fundamental role in cell cycle control and cell growth [27], protein and lipid synthesis [7,12], lipid accumulation and adipogenesis [28,29]. Thus, persistently overactivated mTORC1 signaling stimulates weight gain, increases body mass, and fat mass [14,29,30].

Basically, there are five major pathways, that activate mTORC1: 1) the presence of growth factors such as insulin and IGF-1 [3,6,7,11,12], 2) sufficient cellular energy (glucose, ATP) [31,32], 3) the availability of amino acids, predominantly essential branched-chain amino acids (BCAAs) such as leucine [5-10,33], 4) the presence of glutamine for cellular leucine uptake and glutaminolysis-mediated activation of mTORC1 [34-36], and 5) the availability of saturated fatty acids, especially palmitic acid [37].

### Milk provides all signals for mTORC1-activation

Mammalian evolution relies on lactation and its secretory end-product milk, required and sufficient for postnatal growth. Milk is not just a simple nutrient, but represents a sophisticated postnatal endocrine system providing all signals that are required to activate mTORC1 of the milk recipient, physiologically the newborn mammal [2].

### Essential branched-chain amino acids activate mTORC1

**Figure 1:**
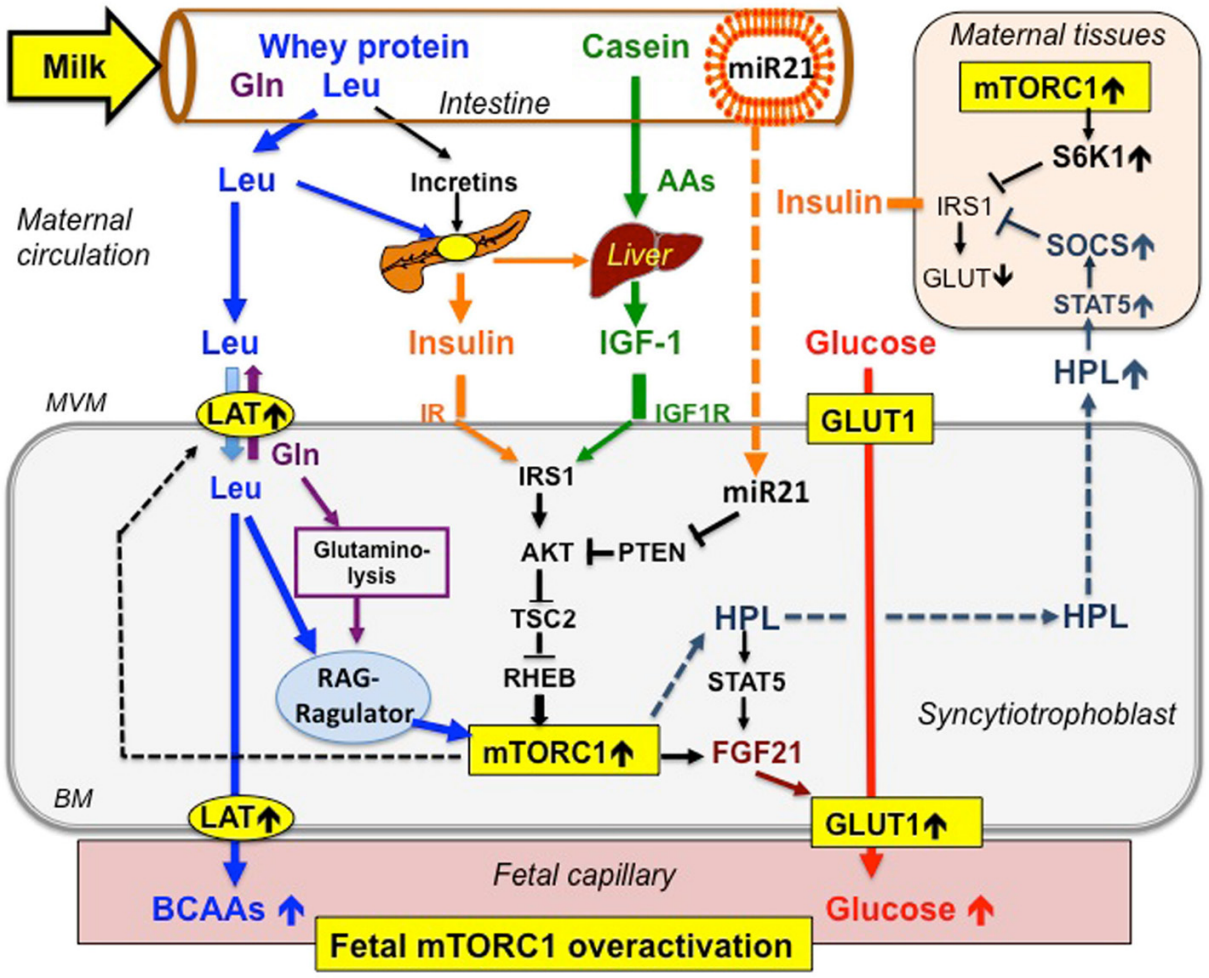
**Interactions between milk and trophoblast mTORC1 signaling.** Leucine (Leu) and glutamine (Gln) derived from whey protein hydrolysis increase serum insulin. Milk casein increases serum IGF-1. Leucine, insulin and IGF-1 stimulate trophoblast mTORC1. Milk consumption is associated with placental weight gain, which is related to increased serum levels of human placental lactogen (HPL). HPL via STAT5/SOCS signaling as well as increased milk-mediated mTORC1/S6K1 signaling induce insulin resistance enhancing the glucose gradient to the fetus. Increased trophoblast mTORC1 and HPL stimulate placental expression of FGF21 upregulating GLUT1. Milk consumption during pregnancy exaggerates glucose transfer to the fetus. Trophoblast mTORC1 stimulates the expression of L-type amino acid transporters (LAT) (dotted line). Thus, milk intake during pregnancy overstimulates diaplacental flux of glucose and BCAAs promoting mTORC1-driven fetal overgrowth. Bovine exosomal microRNA-21 (miR-21) reaches maternal circulation and may thus decrease trophobast PTEN expression thereby enhancing trophoblast mTORC1 signaling.

Milk proteins provide highest amounts of essential BCAAs, especially leucine [38]. Leucine plays a pivotal role for activating mTORC1 (Figure 1) [33]. Of all animal proteins, whey proteins contain the highest amount of leucine (14%) [38], and in comparsion to meat (8% leucine), whey proteins undergo fast intestinal hydrolysis, thus operate like an i.v. amino acid infusion [39-42].

### Glutamine activates mTORC1

Milk protein (8.09 g glutamine/100 g) in comparison to beef protein (4.75 g glutamine/100 g) provides 70% more glutamine [43]. Glutamine is an important activator of mTORC1 via its function as a gatekeeper for cellular leucine uptake and via its precursor function in the glutaminolysis pathway that activates mTORC1 (Figure 1) [44-46]. Leucine is an allosteric activator of glutamate dehydrogenase (GDH), the key-regulating enzyme of the glutaminolysis pathway [44,45]. The interplay of glutamine and leucine maximizes the flux through GDH in pancreatic β-cells, which is important for mTORC1-S6K1-dependent insulin secretion [46].

### Insulin activates mTORC1

Milk stimulates insulin secretion (Figure 1) [2]. The *insulinemic index* of whole cow’s milk (148 ± 14) and skim milk (140 ± 13) is much higher than the glycemic indices of whole milk (42 ± 5) and skim milk (37 ± 9), respectively [47,48]. Fast hydrolysis and immediate intestinal absorption of insulinotropic amino acids of the whey protein fraction of cow’s milk raises insulin levels to much higher magnitudes than intestinal digestion of structural proteins such as beef (*insulinemic index*: 51) [47,48]. The major insulinotropic protein fraction of cow’s milk is the whey protein fraction [49]. Whey-derived leucine and other whey-derived amino acids stimulate incretin secretion of enteroendocrine K- and L-cells [50-54]. Additionally, whey-derived amino acids directly exert inulinotropic effects on pancreatic β-cells [55-57]. Milk protein consumption in comparison to meat protein intake thus results in hyperinsulinemia [58].

### Insulin-like growth factor-1 activates mTORC1

A meta-analysis confirmed that continued milk consumption increases serum levels of insulin-like growth factor-1 (IGF-1) [59]. The *European Prospective Investigation into Cancer and Nutrition* confirmed a relationship between milk intake in 2 109 European women with increased IGF-1 serum levels [60]. A 20% increase in serum IGF-1 levels has been observed in prepubertal children previously not used to milk consumption after a daily intake of 710 mL of milk for 4 weeks [61]. A recent study including 193 overweight adolescents aged 12–15 years drank either 1 L/day of skimmed milk, whey, casein or water for 12 weeks. All milk-based-drinks contained 35 g milk protein/L. IGF-1 significantly increased with skimmed milk and tended to increase with casein compared to the pre-test control group [62]. Casein in comparison to whey protein has been shown to differentially enhance hepatic IGF-1 synthesis [49]. Notably, per capita cheese consumption, the major dairy source of casein, increased in Germany from 5 kg in 1950 to 24.4 kg in 2013 [63].

### Palmitic acid activates mTORC1

Cow’s milk contains about 3.5 to 5% total lipid. About 98% of the lipid is composed of triacylglycerol, transported in milk fat globules [64]. The major fatty acid of total fatty acids of milk lipids is palmitate (C16:0) with 32.3 wt% [64,65]. Palmitate like BCAAs activates mTORC1 [37].

Thus milk, the promoter of postnatal growth of mammals, activates mTORC1 of the milk recipient either by transfer or induction of critical mTORC1 activating signals (Figure 1).

It is the intention of this review to demonstrate that milk consumption during pregnancy increases weight trajectories of the growing human fetus promoting increased birth weight, a well-known risk factor for the development of diseases of civilization.

### Milk consumption and pregravid maternal weight

Prepregnancy maternal overweight and obesity are risk factors promoting fetal overnutrition and macrosomia [18-26]. Obesity is associated with enhanced TORC1 signaling [14-16]. In obesity serum levels of insulin, BCAAs, and free palmitate are increased [66-69]. In obese children additional supply of leucine resulted in excessive hyperinsulinemia [70]. Elevated serum levels of BCAAs in children and adolescents have been identified as predictors of insulin resistance [69]. Notably, milk protein but not meat protein consumption induced hyperinsulinemia and insulin resistance [58]. In obesity and states of insulin resistance, palmitate serum levels are significantly elevated [71-73]. Milk-mediated stimulation of mTORC1 increases the phosphorylation of the major mTORC1 substrate, S6 kinase 1 (S6K1) [14]. Overactivated S6K1 via phosphorylation of insulin receptor substrate-1 (IRS-1) is a pivotal mechanism that induces insulin resistance [74,75].

There is substantial evidence that milk consumption in children increases linear growth and body mass index (BMI) [76-78], increases BMI in adolescents, and adults [79-81]. Noteworthy, a recent meta-analysis of Chen *et al*. [82] including 29 randomized controlled trials found no significant effects of total dairy intake on body weight and body fat [82]. Notably, this study did not differentiate between milk and other processed milk products. The study of Abreu *et al.* [83] reported a protective association between dairy product consumption and abdominal obesity among Azorean boys. However, this study using a self-administered semiquantitative food frequency questionnaire (categorizing < 2 and ≥ 2 servings per day) did not discriminate between the effects of milk consumption versus other dairy products and did not provide quantitative dose–response data on daily milk intake. By using the same semiquantitative food frequency questionnaire methodology categorizing the number of servings/day the authors reported an inverse association between milk intake and both BMI and body fat in 583 Azorean girls but not in 418 Azorean boys [84]. In contrast, Arnberg *et al.* [80] investigated 203 overweight adolescents with a BMI of 25.4 ± 2.3 kg/m^2^ (mean ± SD), who received an additional daily amount of 35 g milk protein either as 1 L/day of skim milk, whey, or casein, or water as a control for 12 weeks. BMI-for-age Z-score was greater at 12 weeks in the skim milk, whey, and casein groups compared with baseline and the control groups [80]. Remarkably, the National Health and Nutrition Examination Survey (NHANES) data from 1999 to 2004 including 1,493 children of age 2–4 years and 2,526 children of age 5–10 years reported an association for the highest quartiles of milk consumption and BMI in contrast to other dairy products, which had no effect on BMI [77]. It is of critical concern that increased prepregnancy BMI represents an important risk factor for increased birth weight of the offspring [21,22,25].

### Milk consumption and gestational weight gain

Women with large for gestational age (LGA) newborns had an increased BMI before pregnancy (25 kg/m^2^), an increased gestational weight gain of 19.0 kg in comparison to women with a normal BMI before pregnancy of 22.4 kg/m^2^ exhibiting a gestational weight gain of 15.8 kg, respectively [85]. Intriguingly, pregnant women gaining excessive weight in comparison to women with optimal weight gain reported a twofold intake of dairy products of about 200 g/day [86]. Of all dairy products, the strongest predictor of increased maternal weight gain during the last trimester of pregnancy was milk [86]. Thus, milk consumption during pregnancy may increase gestational weight gain.

### Milk consumption and placental weight

Data from 50 117 mother-infant pairs of the *Danish National Birth Cohort* collected from 1996–2002 showed a placental weight increase across the whole range of milk intake [87]. A linear increment of placental weight from 13.3 g (0–1 glass of milk/day) to 26.4 g (>6 glasses of milk/day) (p < 0.001) has been reported [87]. A prospective study in India reported that the frequency of milk consumption at 18th week of gestation was positively associated with an increase of placental weight [88].

A milk-induced increase in placental weight may not only raise the nutrient transfer to the fetus but may also increase the amount of placenta-derived growth hormones that impair maternal insulin sensitivity, thereby enhancing maternal blood glucose levels leading to fetal overgrowth and increased birth weight. In fact, maternal blood levels of human placental lactogen (HPL) are correlated with placental weight [89-91], and fetal weight [91-94], respectively. *CSH1*, the predominant transcript of HPL, is increased in placentas of LGA pregnancies [95]. A link between fetal growth velocity in the second half of the pregnancy and maternal serum HPL levels has been demonstrated [96]. In LGA newborns the expression of *CSH1-1*, *CSH2-1*, and *CSHL1-4* mRNA transcripts in placenta was significantly increased compared with appropriate for gestational age (AGA) newborns [85]. Women with LGA newborns had an increased BMI before pregnancy (25 kg/m^2^), an increased gestational weight gain (19 kg), and increased placental weight (777.6 g) compared to AGA newborns associated with a normal maternal BMI before pregnancy of 22.4 kg/m^2^, a gestational weight gain of 15.8 kg, and a placental weight of 650 g, respectively [85].

### mTORC1 promotes placental nutrient transfer

The placenta is the nutrient and endocrine system controlling prenatal mTORC1 signaling for appropriate fetal growth [97,98]. The syncytiotrophoblast, which highly expresses mTOR [99], represents the transporting epithelium and the primary endocrine cell of the human placenta and functions as an mTORC1-dependent nutrient sensor that plays a unique role in the regulation of fetal growth [100]. It has been demonstrated in cultured primary human trophoblast cells that mTORC1 is regulated by glucose, amino acids, and growth factors [101]. mTORC1 is a positive regulator of placental system A and system L amino acid transporters, suggesting that trophoblast mTORC1 modulates amino acid transfer across the placenta [100]. Trophoblast mTORC1 activation increases the cell surface density of amino acid transporters and thus links maternal nutrient availability and growth factor signaling to fetal growth by modulating the mTORC1-mediated flux of amino acids across the placenta, a mechanism that finally results fetal overgrowth (Figure 1) [100].

Activation of placental mTORC1 signaling has been observed in association with maternal obesity [102]. In female Albino Wistar rats, maternal overweight increased placental mTOR and fetal growth [103]. Obesity is associated with elevated circulating levels of BCAAs, free palmitate, hyperinsulinemia, and insulin resistance [66-70,104]. Obviously, the metabolomics of obesity with enhanced nutrient and hormonal signals overstimulate trophoblast mTORC1 activity. In fact, in obese women giving birth to LGA newborns, the activity of placental insulin/IGF-1 and mTORC1 signaling was positively correlated with birth weight [103].

In contrast, mTORC1 in the human placenta is downregulated in restricted fetal growth [99]. Furthermore, in pregnant baboons maternal nutrient restriction downregulated placental mTOR, insulin/IGF-1 signaling and nutrient transporters [105].

### Milk intake and maternal insulin resistance

Maternal insulin resistance is a physiologic adaptation of pregnancy that limits maternal glucose uptake to ensure an adequate supply of glucose that is shunted to the growing fetus. Hyperinsulinemia and insulin resistance start to develop in the second half of pregnancy and are induced by the placenta-derived growth hormones, placental growth hormone (PGH) and human placental lactogen (HPL). The somatogenic and lactogenic hormones of the placenta and maternal pituitary gland integrate the metabolic adaptations of pregnancy with the demands of fetal and neonatal development. Dysregulation of placental growth hormones in pathologic conditions of pregnancy adversely affects fetal growth and postnatal metabolic function [106]. In addition to promoting growth of maternal tissue, PGH induces maternal insulin resistance and thereby facilitates the mobilization of maternal nutrients for fetal growth. HPL and prolactin increase maternal food intake by induction of central leptin resistance and promote maternal β-cell expansion and insulin production [106]. Remarkably, milk consumption during pregnancy increased placental weight [87,88], which has been associated with increased maternal serum levels of HPL [89-91]. PGH, which activates the maternal GH receptor (GHR), and HPL, which activates the maternal prolactin receptor (PRLR) both induce signal transducer and activator of transcription 5 (STAT5) [107]. STAT5 promotes the expression of suppressor of cytokine signaling proteins (SOCS) [107]. It is well known that HPL stimulates the Janus-activated-kinase-2 (JAK2)/STAT5 signaling pathway [108-110]. HPL induces SOCS1 and SOCS2 [111]. SOCS1, SOCS3, SOCS6 and SOCS7 are negative regulators of insulin signaling by binding to the insulin receptor (IR), blocking access of signaling intermediates and inhibiting IR tyrosine kinase activity, leading to a reduction of IR-directed phosphorylation of IRS-1 and its downstream events, and by targeting IRS-1 and IRS-2 for proteasomal degradation [112-114]. Increased PGH and HPL signaling via upregulated SOCS expression thus induces SOCS-mediated insulin resistance (Figure 1) [112-115].

Overstimulated mTORC1 signaling activates S6K1 [7,14,116,117], which reduces insulin signaling by inhibitory phosphorylation of IRS-1 [116,117]. BCAA-mediated insulin resistance is explained by enhanced activation of S6K1 [117-124]. In fact, high intake of milk, but not meat, induces insulin resistance in humans [58].

Milk consumption during pregnancy apparently increases the magnitude of maternal insulin resistance 1) by upregulation of placental HPL-SOCS signaling, and 2) by stimulation of maternal mTORC1-S6K1 signaling (Figure 1). Both pathways in a synergistic manner may enhance the magnitude of maternal insulin resistance, thereby increasing the glucose flux to the fetus.

### Milk and FGF21-mediated GLUT1-overexpression

Placental weight gain, which is related to milk consumption during pregnancy, is associated with increased maternal serum levels of HPL [87-91]. HPL activates downstream JAK2/STAT5 signaling [108-110]. Recently, fibroblast growth factor-21 (FGF21) has been related to insulin resistance, type 2 diabetes mellitus, obesity and the metabolic syndrome [125,126]. In comparison to control subjects, plasma FGF21 levels were significantly higher in women with gestational diabetes mellitus (GDM) [127]. Increased mRNA expression of FGF21 has been detected in the placenta of women with GDM [128]. Notably, the FGF21 promoter contains three putative STAT5-binding sites [129]. Increased FGF21 production has been observed in late pregnancy in the mouse [130]. Ectopic activation of hepatic mTORC1 in liver-specific *Tsc1* knockout mice resulted in enhanced expression of FGF21 [131]. Intriguingly, overexpression of FGF21 in 3 T3-L1 adipocytes upregulated glucose uptake and increased mRNA expression of glucose transporter 1 (GLUT1) [132]. GLUT1 is the primary glucose transporter isoform in the human placenta that increases its expression over gestation [133]. GLUT1 has been localized to both the maternal facing microvillous plasma membrane (MVM) with threefold higher expression as compared to the basal plasma membrane (BM) [134]. In maternal diabetes, the expression of GLUT1 in the BM has been reported to increase [135,136]. Moreover, increased BM expression of GLUT1 has been associated with high birth weight of large babies of non-diabetic mothers [137].

Thus, milk-mediated overactivation of mTORC1 via placental overexpression of FGF21 and enhanced HPL/STAT5-driven placental expression of FGF21 may overstimulate trophoblast GLUT1 expression that increases the diaplacental flux of glucose to the fetus (Figure 1). Alterations of maternal and placental metabolic signaling by milk consumption during pregnancy may thus explain accelerated fetal growth and increased birth weight.

### MicroRNA-21 and placental, fetal and adipocyte growth

Jiang *et al.* [138] recently reported on aberrant upregulation of microRNA-21 in placental tissue of macrosomia. Importantly, exosomal microRNA-21 is an abundant and consistent microRNA of cow’s milk [139]. Notably, human and bovine microRNA-21 stem-loops are identical (www.mirbase.org). Milk has been proposed to function as a metabolic transfection system by transfer of exosomal microRNAs activating mTORC1 signaling of the milk recipient [2]. Milk’s exosomal microRNA represent milk’s “software” and milk-derived BCAAs milk’s “hardware” for activating mTORC1 signaling [2]. In fact, Baier *et al.* [140] provided evidence that microRNAs of commercial pasteurized cow’s milk are absorbed by adult human subjects in biologically meaningful amounts from nutritionally relevant doses of cow’s milk and affect gene expression of peripheral blood mononuclear cells, HEK-293 kidney cell cultures and mouse livers. Furthermore, they demonstrated that disintegration of milk exosomes by ultrasonication abolished the transfer and uptake of milk-derived microRNAs [140]. Notably, in placental tissues target genes of microRNA-21 were involved in JAK-STAT, PI3K-AKT, and mTOR signaling pathways [138]. It is thus conceivable that milk-derived exosomal microRNA-21 may reach the trophoblast cell and contributes to overactivated trophoblast mTORC1 signaling. Critical targets of microRNA-21 are mRNAs of important tumor suppressor proteins involved in upstream and downstream suppression of mTORC1 signaling such as PTEN [141-144], Sprouty1 and Sprouty2 [145-147], and PDCD4 [148-150]. Moreover, microRNA-21 has been shown to induce the cell cycle promoter cyclin D1 in an mTORC1-dependent manner [151]. Supposed that milk-derived microRNA-21 reaches the trophoblast cells via systemic circulation of the pregnant milk-consuming mother, PTEN suppression could increase insulin/IGF-1/PI3K/AKT signaling, which further augments mTORC1 activation (Figure 1). MicroRNA-21-mediated inhibition of Sprouty1 and 2 would amplify RAS-RAF-MEK-ERK signaling, which additionally suppresses TSC2 and thus raises mTORC1 activity. Furthermore, microRNA-21 could stimulate the initiation of translation by repression of PDCD4, which is a suppressor of translation initiation that inhibits the RNA helicase eIF4A [152]. Both, 4E-BP-1 and PDCD4 are crucial regulatory inhibitors of translation initiation and thus of protein synthesis. Activation of the mTORC1 pathway and its substrate kinase S6K1 results in subsequent phosphorylation of 4E-BP-1 and PDCD4 that promote eIF4E-eIF4G complex assembly and stimulate mRNA translation [151]. Milk microRNA-21-mediated suppression of PDCD4 expression may further amply translation, which enhances placental and finally fetal overgrowth. Furthermore, microRNA-21 promotes adipogenic differentiation and proliferation of human adipose tissue-derived mesenchymal stem cells [153,154], thus promoting fat mass accretion. It has recently been shown that long-term inhibition of microRNA-21 reduced obesity in db/db mice [155].

### Milk consumption and fetal and birth weight

The *Generation R Study*, a population-based prospective cohort study from fetal life until young adulthood in Rotterdam investigated 3 405 mothers during pregnancy [156]. Maternal milk consumption of >3 glasses (450 mL of milk) per day was associated with greater fetal weight gain in the third trimester of pregnancy, which led to an 88 g higher birth weight than that with milk consumption of 0 to 1 glass per day [156]. This association was limited to milk, whereas protein intake from nondairy food or cheese was not associated with increased birth weight. A possible explanation for this finding is the presence of biologically active microRNAs in milk and their absence in processed milk products such as cheese. Compared with the lowest reference category of milk consumption (0–1 glasses/day), maternal milk intakes of > 1–2 glasses/day, 2–3 glasses/day, and > 3 glasses/day were associated with increased fetal weight gain. Fetal weight gain has been estimated by the procedure of Hadlock *et al.* [157]. Milk-mediated differences in fetal weight gain appeared from the 20th week onward, but became most evident in the last part of the third trimester [156], a period that is controlled by HLP signaling.

Worldwide studies confirmed an increase of birth weight in relation to milk consumption during pregnancy (Table 1). A retrospective cohort in Sweden reported a birth weight increase of 75 g and 134 g in the offspring of mothers consuming > 200 mL and 1 L milk daily, respectively [158]. A prospective study in India reported that the frequency of milk consumption at the 18th week of gestation was positively associated with birth weight, birth length, and head circumference [88]. According to a prospective study in Canada, maternal daily consumption of an additional 237 mL of milk was associated with a 41 g increase in offspring birth weight [159]. A prospective Australian study in 557 mothers reported that protein intake from dairy products was associated with a higher birth weight of the offspring [160]. In a randomized controlled trial of 72 adolescent pregnant mothers in the USA, 25 mothers were counseled to consume > 4 servings of dairy products a day, which resulted in a 240 g higher birth weight compared to the control group [161]. According to a systematic literature review, the majority of studies reported positive associations between milk and/or dairy consumption and birth weight-related outcomes [162].

**Table 1:** **Milk intake increases prepregnancy, gestational, placental, fetal, and birth weight**

**Effect of milk intake**	**Outcome**	**Studies [Ref.]**
Prepregnancy weight	Increase	Randomized intervention study, Denmark [80]
Gestational weight gain	Increase	Observational study, Iceland [86]
Placental weight	Increase	Danish National Birth Cohort, Denmark [87]
	Increase	Pune Maternal Nutrition Study, India [88]
Fetal weight	Increase	Generation R Study, Netherlands [156]
Birth weight	Increase	Generation R Study, Netherlands [156]
	Increase	Observational study, Sweden [158]
	Increase	Pune Maternal Nutrition Study, India [88]
	Increase	Observational study, Canada [159]
	Increase	Prospective observational study, Australia [160]
	Increase	Randomized controlled trial, USA [161]
	Increase	Systematic literature review, Norway [162]

## Conclusions

We provided literature evidence supported by translational research that milk consumption increases pregravid, gestational, placental, fetal, and birth weight, respectively (Table 1). The *Mater-University Study of Pregnancy and Its Outcomes* demonstrated that maternal BMI in comparison to paternal BMI is associated with the BMI of the offspring at ages 5 and 14 years [22]. Based on these data, Lawlor *et al.* [22] proposed the *fetal overnutrition hypothesis of obesity* and concluded that nutrient-dependent programming effects during fetal life are responsible for the development of obesity [22].

The central hub of nutrient sensing, growth regulation and anabolism is the kinase mTORC1, which is upregulated in obese subjects and by milk consumption [2,14-17]. Milk is an evolutionary feeding and anabolic programming system controlled by the lactation genome that regulates mTORC1-dependent postnatal growth by donation of mTORC1-activating essential BCAAs and exosomal microRNAs [2,140]. The placenta is the natural feeding and programming system controlling mTORC1-dependent fetal growth. No other gravid mammal is simultaneously exposed to lactation-driven as well as placenta-driven mTORC1 signaling, except human beings since the Neolithic revolution boosted after the widespread distribution of refrigerators in the early 1950’s allowing daily access to bovine milk. From an anthropological perspective, Wiley [13] concluded that milk consumption by humans is a novel behavior that increases BMI and may induce long-lasting adverse effects on human health. In fact, our evidence underlines that milk consumption increases prepregnancy BMI [79-81], gestational [85,86], placental [87,88], fetal [156], and birth weight [156,158-162], respectively. Notably, increased birth weight, is a risk factor for the development of mTORC1-driven diseases of civilization [163-171]. The magnitude of fetal and postnatal mTORC1-signaling apparently determines lifelong axes of metabolic, hypothalamic and immunological programming [172-176].

Intrauterine overnutrition affects the risk of obesity [177-180]. High maternal plasma concentrations of glucose, amino acids and free fatty acids have been implicated to result in permanent changes in appetite control, neuroendocrine functioning, and energy metabolism in the developing fetus, thus leading to obesity later in life [176-179]. Milk consumption provides abundant BCAAs and palmitate, stimulates insulin/IGF-1 signaling, and provides abundant exosomal microRNAs that in a synergistic manner may overstimulate trophoblast mTORC1 activity (Figure 1). Overactivated trophoblast mTORC1 signaling finally explains 1) increased expression of mTORC1-dependent amino acid transporters with enhanced diaplacental flux of amino acids, 2) increased HPL synthesis with STAT5-promoted induction of maternal insulin resistance thus increasing the glucose flux to the fetus, and 3) increased STAT5/FGF21- and mTORC1/FGF21-driven trophoblast GLUT1 expression promoting diaplacental glucose transfer to the fetus. Accordingly, fetal cells obtain an excessive supply of glucose and BCAAs. Finally, BCAAs that reach fetal cells overactivate fetal mTORC1 signaling promoting fetal overgrowth [180].

When overactivated mTORC1 signaling persists during the postnatal period by the introduction of artificial high protein formula feeding, lifelong deviations of mTORC1-dependent metabolic, neuroendocrine and immunological programming may result [181,182]. In this regard, the worst scenario for mTORC1-dependent perinatal malprogramming is an obese mother, who increases milk consumption during pregnancy, and provides excessive protein by artificial formula feeding [182]. High milk intake during pregnancy and high protein formula feeding may synergistically enhance perinatal mTORC1 signaling explaining the *fetal overnutrition hypothesis* and the *early protein hypothesis* [22,174,182]. These two hypotheses converge to a *perinatal mTORC1-overactivation hypothesis*, explaining the adverse effects of increased milk-mediated mTORC1 signaling during the pre- and postnatal period of metabolic programming.

Current dietary recommendations for pregnant women intend to assure sufficient supply of calcium and high quality proteins for the growing fetus. However, there is more and more concern about milk’s role as a source of calcium. According to the recent opinion of *Harvard School of Public Health* milk isn’t the only, or even best, source of calcium [183]. There are non-dairy foods including leafy green vegetables, broccoli, beans and tofu that supply high amounts of calcium. These calcium-rich food alternatives have a significant advantage in comparison to milk: they do not overstimulate mTORC1 signaling and most importantly do not transfer biologically active exosomal microRNAs [141].

Therefore, we suggest to re-evaluate dietary recommendations for pregnant women. We appeal to the medical community to define save upper limits for milk consumption during pregnancy, especially for those women who enter gravity with increased BMI. Whereas boiling of milk destroys milk’s bioactive microRNAs [184], boiling has no effect on milk-BCAA-mediated mTORC1 activation. Future randomized-controlled clinical studies are needed to better study the effect of dietary interventions based on milk consumption’s difference during pregnancy, especially in women who enter pregnancy with overweight or obesity, and the risk of increased birth weight [185].

## Abbreviations

AAs: Amino acids
AGA: Appropriate for gestational age
BCAA: Branched-chain amino acid
BM: Basal membrane
BMI: Body mass index
4E-BP-1: Eukaryotic translation initiation factor 4E-binding protein 1
FGF21: Fibroblast growth factor 21
GDH: Glutamate dehydrogenase
GDM: Gestational diabetes mellitus
GHR: Growth hormone receptor
GIP: Glucose-dependent insulinotropic polypeptide
GLP-1: Glucagon-like peptide-1
HPL: Human placental lactogen
IGF-1: Insulin-like growth factor-1
IGF1R: Insulin-like growth factor-1 receptor
IR: Insulin receptor
IRS: Insulin receptor substrate
JAK: Janus-activated kinase
PI3K: Phosphoinositide-3 kinase
LAT: L-type amino acid transporter
Leu: Leucine
LGA: Large for gestational age
MicroRNA: Micro-ribonucleic acid
mTORC1: Mechanistic target of rapamycin complex 1
MVM: Microvillous plasma membrane
PGH: Placental growth hormone
PRLR: Prolactin receptor
PTEN: Phosphatase and tensin homolog
RHEB: RAS homolog enrich in brain
S6K1: Ribosomal protein S6 kinase, 70-kD kinase 1
SOCS: Suppressor of cytokine signaling
TSC2: Tuberin
